# Multi-Criteria Decision Analysis to Prioritize People for COVID-19 Vaccination When Vaccines Are in Short Supply

**DOI:** 10.3389/frhs.2022.760626

**Published:** 2022-04-26

**Authors:** Hend Chaker Masmoudi, Amal Rhili, Imen Zamali, Ahlem Ben Hmid, Melika Ben Ahmed, Myriam Razgallah Khrouf

**Affiliations:** ^1^Faculty of Pharmacy of Monastir, University of Monastir, Monastir, Tunisia; ^2^Department of Histology and Cytogenetics, Institute Pasteur of Tunis, Tunis, Tunisia; ^3^Department of Clinical Immunology, Pasteur Institute of Tunis, Tunis, Tunisia; ^4^Faculty of Medicine of Tunis, University of Tunis El Manar, Tunis, Tunisia; ^5^Direction de la Pharmacie et du Médicament, Tunis, Tunisia

**Keywords:** COVID - 19, MCDA (multi-criteria decision analysis), vaccination, prioritization, criteria, scoring system

## Abstract

COVID-19 pandemic underscored the need for a rapid tool supporting decision-makers in prioritizing patients in the immediate and overwhelming context of pandemics, where shortages in different healthcare resources are faced. We have proposed Multi-Criteria Decision Analysis (MCDA) to create a system of criteria and weights to prioritize uses of COVID-19 vaccines in groups of people at significantly higher risk of severe COVID-19 disease or death, when vaccines are in short supply, for use in Tunisia. The prioritization criteria and the levels within each criterion were identified based on available COVID-19 evidence with a focus on the criteria selected by Tunisian scientific committees. To determine the weights for the criteria and levels, reflecting their relative importance, a panel of frontline physicians treating COVID-19 were invited to participate in an online survey using 1,000 minds MCDA software (www.1000minds.com) which implements the PAPRIKA (Potentially All Pairwise RanKings of all possible Alternatives) method. Ten criteria and twenty-three levels have been selected for prioritizing the uses of COVID-19 vaccines in groups of people at significantly higher risk of severe disease or death. Among the invited physicians, sixty have completed the survey. The obtained scores were, in decreasing order of importance (mean weights in parentheses, summing to 100%). Obesity (16.2%), Age (12.7%), Chronic pulmonary diseases (10.8%), Chronic cardiovascular conditions (10.3%), Bone marrow or organ transplantation (10.1%), Immunodeficiency or Immunosuppression (9.6%), Diabetes (9%), Renal failure (8.4%), evolutive cancer (6.9%), and high blood pressure (6%). MCDA-based prioritization scoring system comprising explicit criteria and weights provides an adaptable and multicriteria approach that can assist policy-makers to prioritize uses of COVID-19 vaccines.

## Introduction

MCDA approach is a rapid and innovative tool to create a “scoring” or “points” system for prioritizing patients for elective health service (1). In public health systems, an optimal prioritization to serve the most urgent patients first can be needed for different applications. This aims for a transparent, equitable, and accountable allocation of limited resources. For example, MCDA has been used for coronary artery bypass graft in New Zealand (1), or solid organ transplantation among patients waitlisted for transplantation (2).

In the context of pandemics, the increase of demand for different health services and resources underscore the need for a rapid tool supporting decision-makers in planning public health strategies and targeting priority groups. During the COVID-19 pandemic, MCDA has been applied to prioritize COVID-19 patients for hospital (3) and intensive care admissions (4).

In MENA countries, few pilot studies used the MCDA model in the region for healthcare applications, to create a value-based system to assess innovative/biology drugs in Egypt (5) or to purchase generic medicines, in Kuwait (6), and in the UAE (7). A broader utilization of the MCDA model in the region is considered to increase the consistency and transparency of policy decisions (8).

Severe acute respiratory syndrome coronavirus 2 (SARS-CoV-2) or COVID-19, originated at Wuhan city of China in early December 2019, has spread across the globe with a profound impact on health and the economy. The high burden of Coronavirus disease 19 (COVID-19) morbidity and mortality has led to a large global effort to develop safe and effective vaccines along with public health measures to contain the pandemic. On December 11, 2020, the FDA issued the authorization for emergency use of the first COVID-19 vaccine (9), followed by the authorization for emergency use of several COVID-19 vaccines in various countries.

The global production capacity of pharmaceutical industries, constraints related to technology licensing, and the high demand for the vaccine, have limited SARS-CoV-2 vaccine supplies worldwide, especially impacting the access of low- and middle-income countries (LMICs) to vaccines. Hence, a huge effort was required to optimize resource deployment in the context of a vaccine shortage.

The WHO Strategic Advisory Group of Experts on Immunization (WHO SAGE) provided a values framework for Allocation and Prioritization of COVID-19 Vaccination and a prioritization roadmap to support countries in planning public health strategies (10). Groups with comorbidities or health states determined to be at significantly higher risk of severe disease or death are among groups to prioritize for COVID-19 vaccination. Each country needed to adopt and further adapt SAGE guidelines depending on the local context, the size of the target groups, vaccine supply, and the evolving knowledge about COVID-19 and vaccines.

Tunisia, in alignment with SAGE values and the suggested prioritization roadmap, addressed a national strategic vaccination plan. Plan's first stage aimed to protect healthcare professionals as essential workers and, second, to reduce the mortality and morbidity burden by prioritizing the elderly and people with comorbidities. Hence, the first supplies of vaccines received were allocated to healthcare professionals, then to the elderly and people with comorbidities to ensure a prioritization based on utilitarian and egalitarian principles, respectively.

Age and specific pre-existing conditions have been proven to be prominent risk factors for COVID-19 morbidity and mortality (11–14). Authorities and health regulatory agencies, as in France (15), the UK (16, 17), and in the USA (18) enumerated the comorbidities that should be considered in their relative vaccination plans. The relative risk of pre-existing comorbidities to COVID-19 morbidity and mortality is variable and the co-existence of more than one condition increases this risk (19). The size of the group of people vulnerable to COVID-19 and to prioritize for vaccination may vary significantly between countries, depending on the whole size of the population, the size of the elder population, and the prevalence rate of comorbidities.

Tunisian National Scientific Committees have considered a scoring system providing an adaptable and multicriteria approach of prioritization of higher risk of morbidity and mortality groups. The criteria of prioritization were decided by the National Committees and were set in e-vax, a dedicated national platform for the registration for the whole population willing to be vaccinated (https://www.evax.tn/).

Our research work aims to use the MCDA model to support decision makers in creating a scoring-based system to prioritize vulnerable people for COVID-19 vaccination, and to reduce therefore COVID-19 morbidity and mortality when the vaccine is in shortage.

## Materials and Methods

To create the MCDA system for prioritizing people for COVID-19 vaccination, we followed the guidelines of the International Society for Pharmacoeconomics and Outcomes Research (ISPOR) Task Force for MCDA application in Prioritizing patients' access to healthcare (20, 21).

**Figure 1:**
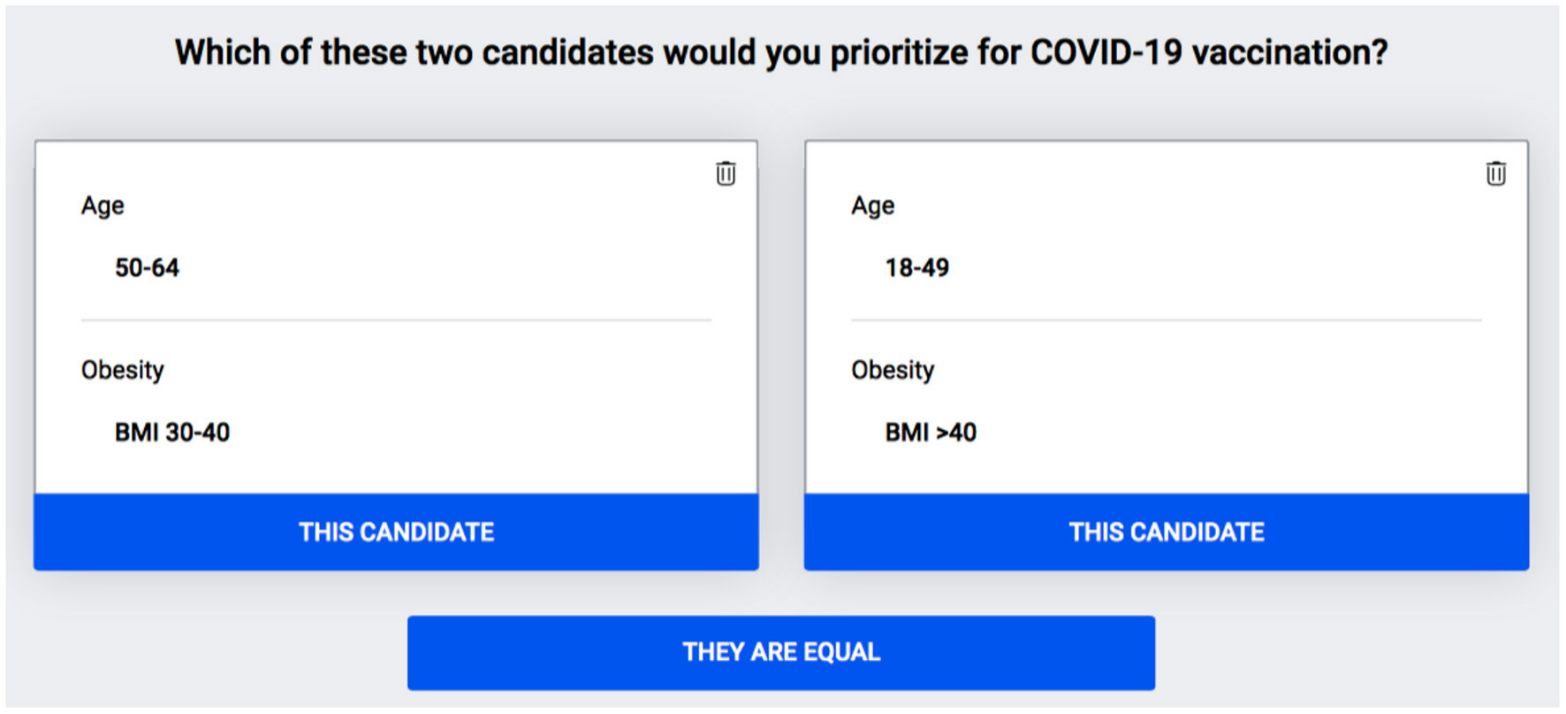
An example of a trade-off question presenting a combination of two levels of two criteria. Age in years, BMI, Body Mass Index.

First, to identify the prioritization criteria and the levels within each criterion, we reviewed the literature, consulted Tunisian decision-makers, and considered also the criteria included in the Evax platform, the national Tunisian platform for COVID-19 vaccination registration. Second, to determine the weights for the criteria and their levels, reflecting their relative importance, a panel of 100 experts and frontline physicians treating COVID-19 in Tunisia were invited to participate in an online survey using 1,000 minds MCDA software (www.1000minds.com) which implements the PAPRIKA (Potentially All Pairwise RanKings of all possible Alternatives) method (22). This software and method have been used in previous studies to prioritize patients for elective surgery (1), non-critical COVID-19 patients for hospital admission (3, 4), critical patients for intensive care (3), and to also create the WHO's priority list for antibiotic-resistant bacteria to help develop new drugs (23). The software shows each participant a series of pairs of combinations of the levels on two criteria at the time, representing two hypothetical candidates for vaccination, and asks: “Which individual would you prioritize for vaccination?” (Figure 1). Each combination involves a trade-off between the two criteria, and the participant's choices reveal how they feel about the relative importance, or ‘weight’, of the two criteria. Each time a participant answers a question, based on all their preceding answers, PAPRIKA adapts with respect to choosing the next question to ask by applying the logical property of ‘transitivity’ – until all possible combinations of the levels on two criteria at the time have been pairwise ranked, either explicitly or implicitly by the participant [For technical details on using PAPRIKA for scoring additive Multi-attribute Value Models please refer to Hansen and Ombler (22)]. Finally, from the participant's explicit pairwise rankings (i.e., answers to the questions) the software uses quantitative methods to derive weights for the levels on each criterion. Obtained Weights for each criterion's level were averaged across all participants to produce mean weights for the group as a whole.

Additional questions were included to collect information on the participants' medical specialties, and their affiliation (public or private institution). The software recorded for each participant the number of questions answered and the time spent to complete the survey.

The experts invited to participate in the survey were deliberately selected to be from different regions in Tunisia and from the various medical specialties working in healthcare settings admitting COVID-19 patients, including intensive care anesthetists, emergency physicians, pulmonologists, infectious disease specialists, internists, endocrinologists, cardiologists, and oncologists, with a focus on the first five cited specialties, as more in charge of COVID-19 severe cases. These experts are all frontline physicians treating COVID-19 patients in Tunisia working mainly in the public sector in national institutions for COVID-19 care and are therefore familiar with dealing with many COVID patients from their various fields of expertise.

Stata 16.1 was used to undertake a one-way analysis of variance for normally distributed variables and the Kruskal-Wallis rank test was run for variables not normally distributed, to test the significance of differences in the criteria's mean weights (*p* < 0.05). We tested the robustness of our model by assessing the Heterogeneity in preferences (weights) among subgroups or sub-specialties of the participants taking part in the present study (20).

## Results

The board of experts from the COVID-19-Tunisian scientific committees identified 10 criteria for prioritizing vulnerable populations for vaccination, as part of the first goal of the national vaccination program. The criteria, with their levels in parentheses, are: (1) Age (<50, 50–64, 65–75, >75 years); (2) Body Mass Index (BMI <30, BMI 30–40, BMI >40); (3) Diabetes (No, Yes); (4) Chronic pulmonary diseases (No, Yes); (5) Chronic cardiovascular diseases (No, Yes); 6. Renal failure (No, Yes); (7) Bone marrow or Organ Transplantation (No, Yes); (8) Immunodeficiency or Immunosuppression related to treatment or condition (No, Yes); (9) Evolutive Cancer (No, Yes); (10) High Blood pressure (No, Yes).

Sixty physicians completed the survey out of 100 invited participants. The mean number of pairwise-ranking questions answered by each participant was 31, taking 10 min 22 s in total on average. The characteristics of the survey participants are summarized in Table 1.

**Table 1:** The characteristics of the survey participants.

**Participants**	**Number = 60**
**Gender**	
Male	25 (41.6%)
Female	35 (58.4%)
**Sector**	
Public	50 (83.3%)
Private	10 (16.7%)
**Specialties**	
Infectious disease specialists and internists	21 (35%)
Intensive care anesthesists	13 (21.7%)
Pulmonologists	9 (15%)
Emergency physicians	4 (6.7%)
Endocrinologists	5 (8.3%)
Oncologists	4 (6.7%)
Cardiologists	2 (3.3%)
Nerurologists	2 (3.3%)

**Figure 2:**
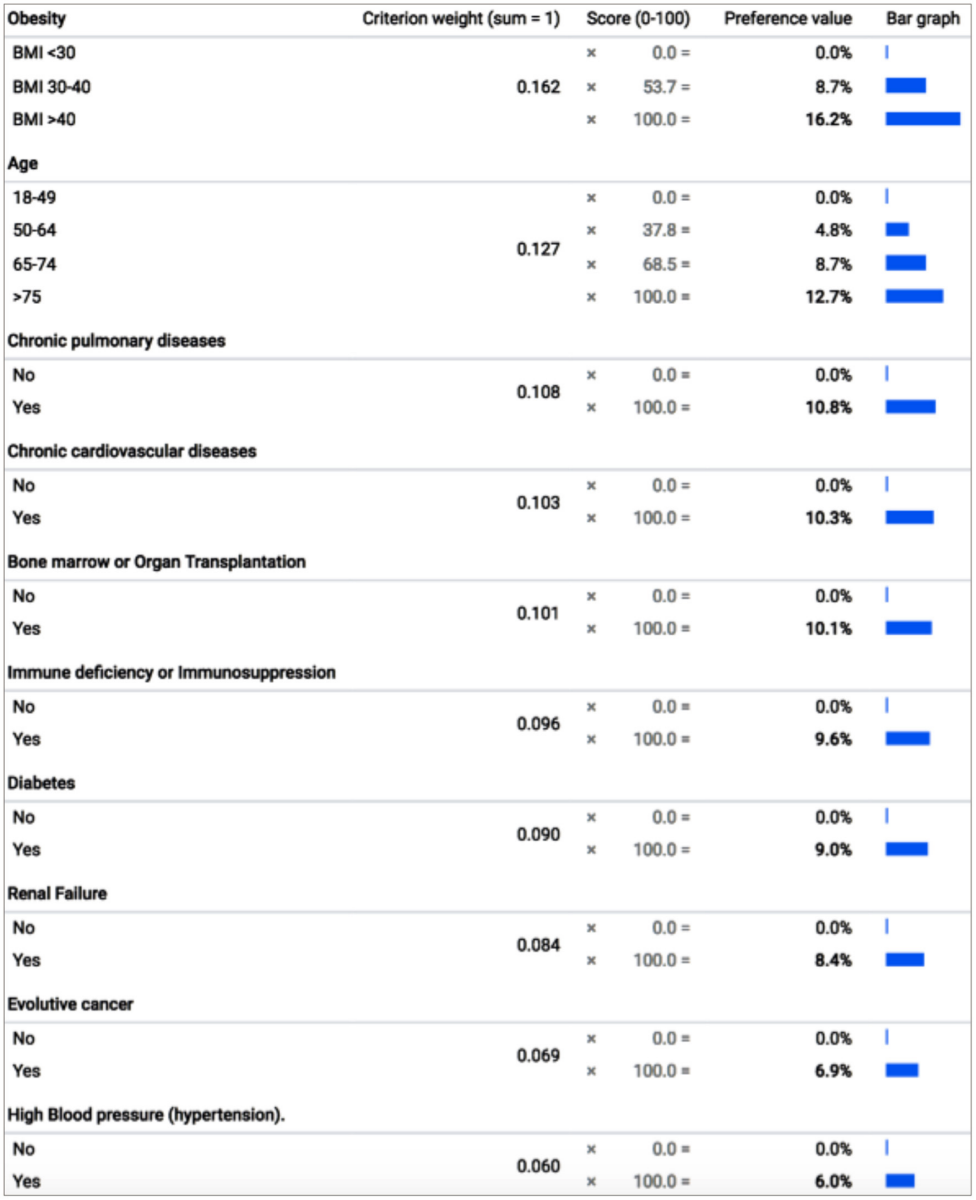
Criteria weights (means) for prioritizing people for COVID-19 vaccination. The bolded values sum to 1 (100%), where the preference values are the criterion weights multiplied by the scores and the bar graph shows the relative importance of every level of each criterion. BMI, Body Mass Index.

**Figure 3:**
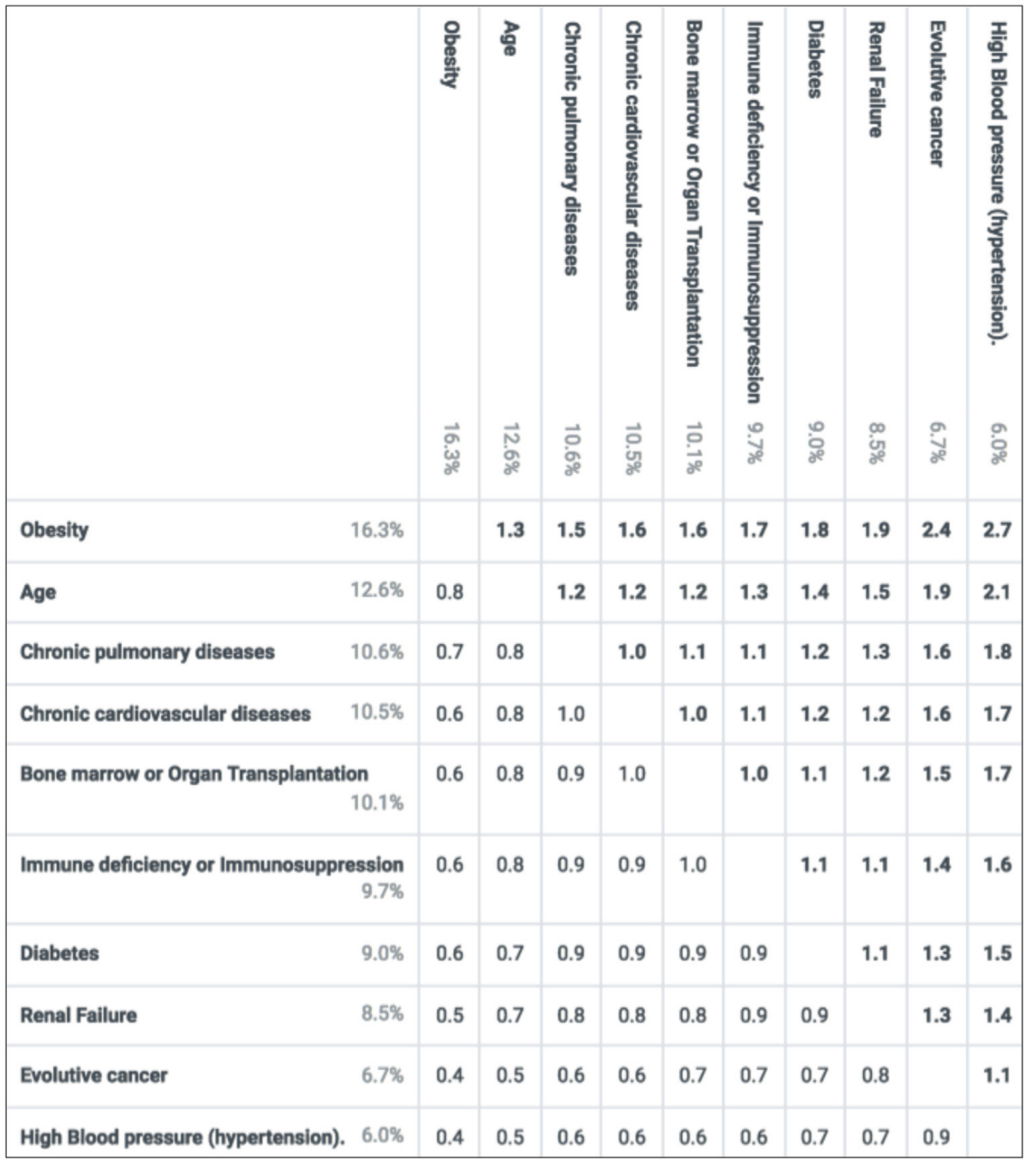
Relative importance of the criteria. Based on the mean preference values, each number in the table is a ratio corresponding to the importance of the criterion on the left relative to the criterion at the top. The ratios are obtained by dividing the left preference values by the top preference values.

Mean weights of the criteria and their levels are reported in Figure 2. The scores of Age and comorbidities in prioritizing COVID-19 candidates to vaccination, as revealed from the experts' answers and capture of preferences were, Obesity (16.2%), Age (12.7%), Chronic pulmonary diseases (10.8%), Chronic cardiovascular conditions (10.3%), Bone marrow or organ transplantation (10.1%), Immunodeficiency or Immunosuppression (9.6%), Diabetes (9%), Renal failure (8.4%), evolutive cancer (6.9%), and high blood pressure (6%). The weight for the highest-ranked level on a criterion represents the criterion's overall weight (relative to the other criteria, with these weights summing to 100%). Each criterion's lowest level has a value of zero. For the two criteria with more than two levels – age and obesity – the weight for their middle levels is relative to the lowest- and highest-ranked levels, respectively. The relative importance of any pair of criteria, as illustrated in Figure 3, was obtained by dividing the preference value of the left criterion by the preference value of the top criterion. For example, “obesity” was 1.8 times more important than “diabetes” (16.2 vs. 9%), whereas “chronic cardiovascular diseases,” “chronic pulmonary diseases” and “bone marrow or organ transplantation” were almost equally important (their overall weights were very close). As determined by each criterion's overall weight, Figure 3 highlights the high importance placed by the panel on “obesity” and “age,” as, compared to any other criteria, their relative-importance ratio is constantly >1, reaching 1.8–2.7 when compared to “evolutive cancer” and “hypertension,” the least important prioritization criteria, according to the experts, on average. Analysis of variance showed statistically significant differences in the mean scores between several criteria (*p* < 0.05).

We simulated a ranking of 11 hypothetical candidates for vaccination, distinguished by their ratings on the criteria. The ranking is based on the total score obtained by summing their weights (Table 2).

**Table 2:** Total scores and ranking of 11 simulated candidates registering for COVID-19 vaccination.

**Age**	**Diabetes**	**Obesity**	**Immune deficiency or Immunosuppression**	**Renal Failure**	**Chronic pulmonary diseases**	**Chronic cardiovascular diseases**	**High Blood pressure (hypertension)**	**Evolutive cancer**	**Bone marrow or Organ Transplantation**
>75	Yes	BMI 30–40	Yes	No	No	No	No	No	No
>75	No	BMI 30–40	No	No	No	Yes	No	No	No
50–64	No	BMI 30–40	No	No	Yes	No	No	Yes	No
50–64	No	BMI >40	No	No	No	No	Yes	No	No
50–64	No	BMI >40	No	No	No	No	Yes	No	No
65–74	Yes	BMI 30–40	No	No	No	No	No	No	No
65–74	Yes	BMI <30	No	Yes	No	No	No	No	No
65–74	No	BMI 30–40	No	No	No	No	Yes	No	No
18–49	No	BMI >40	No	No	No	No	Yes	No	No
65–74	No	BMI 30–40	No	No	No	No	No	No	No
18–49	No	BMI 30–40	No	No	No	No	No	Yes	No

*The total score of candidates were obtained by summing the weights of their relative level of criteria. BMI, Body Mass Index; BM, Bone Marrow*.

We tested the robustness of our model by assessing the Heterogeneity in preferences (weights) among subgroups of the participants taking part in the present study (20). Results are summarized in Table 3.

**Table 3:** Average of selected levels of criteria among participant subgroups.

	**Obesity: BMI >40**	**Age: >75 ys**	**Chronic cardiovascular disease**	**Diabetes**	**Chronic pulmoray disease**
Infectious disease specialists and internists	14.69%	12.52%	10.14%	9.18%	11.77%
Pulmonologists	16.71%	12.69%	9.80%	9.04%	8.64%
Intensive care anesthesists	18.88%	14.68%	9.29%	8.52%	10.73%
Emergency physicians	16.06%	10.14%	9.29%	11.17%	9.26%
Endocrinologists	16.32%	14.52%	14.59%	7.84%	15.26%
Oncologists	12.00%	16.25%	10.37%	7.62%	7.80%
Mean /SD	**15.78/2.3**	**13.47/2.14**	**10.55/2.01**	**8.89/1.27**	**10.58/2.7**

*Levels selected to show the weight among different subgroups of participants are: Obesity (BMI >40), Age >75 years, the existence of chronic cardiovascular disease, the existence of diabetes, the existence of chronic pulmonary disease. SD, Standard Deviation. The bold values used to indicate mean/SD values (to differentiate mean/SD values from subgroups values)*.

## Discussion

COVID-19 pandemic underscores the need for a rapid tool to establish a scoring system for the optimal allocation of available resources. In the context of the limited supply of COVID-19 vaccines, prioritizing candidates that the most vulnerable people receive the vaccine first was one of the main goals of the COVID-19 vaccination program in Tunisia. A scoring system is an adaptable approach for phased reception of anti-COVID-19 vaccines and provides a multi-criteria approach to prioritize candidates for vaccination and ensure high-risk individuals get immunized first.

Besides a person's age, pre-existing conditions predispose people infected with COVID-19 to an unfavorable clinical course and increased risk of intubation and death. Of Tunisia's population of 11.94 million, 2.8 million have at least one underlying condition and 710,000 have at least two comorbidities (http://ghdx.healthdata.org/). Decision-makers in Tunisia declared the main criteria to consider for prioritization: age, diabetes, cardiovascular diseases, chronic pulmonary diseases, kidney failure, immunodeficiency or immunosuppression, transplantation, obesity, evolutive or recent cancer, and hypertension.

These criteria are inclusive of the main factors and comorbidities predisposing to COVID-19 morbidity and mortality that different studies, organizations, and authorities reported as factor risks for severe COVID-19 or mortality (15, 17, 18). As selected by a dedicated Tunisian committee of scientists and decision-makers, these criteria reflect the epidemiological context of Tunisia, and also using them in our study allows us to create a scoring system consistent with the national strategy.

In our study, “obesity” and “age” were found to be the most important criteria for determining people's priority for vaccination. Surprisingly, obesity (BMI >40) has been found as the highest weighted criteria in our score-system, higher the weight associated with age (age >75 years old). It is worth noticing that an Italian panel of 100 physicians weighted obesity (BMI >40) higher than age, and other comorbidities factors when weighting criteria for prioritizing hospital admission of patients affected by COVID-19 in the context of a shortage of hospital beds (3).

In the past, obesity has been strongly correlated with mortality from viral infections such as H1N1 influenza and the previous SARS and MERS coronaviruses causing widespread infections (24). Different studies have reported obesity as a strong predictor of COVID-19 severity. A case-control study in Mexico including 32,583 patients (12,304 cases and 20,279 controls), presenting only one co-morbidity, to determine the independent effect of each comorbidity on Covid-19 susceptibility, found obesity as the strongest predictor factor (25). Interestingly, a study found a significant positive linear association between increasing BMI and admission to intensive care units (ICU) due to COVID-19 with a significantly higher risk for every BMI-unit increase (26). Another study from New York showed that age >65 years and obesity are on two most important predisposing factors leading to hospital admission and critical COVID-19 illness – more than hypertension, diabetes, or cardiovascular diseases (27). Recently, the Center for Disease Control and Prevention (CDC) has issued an updated list of underlying medical conditions associated with higher risk for severe COVID-19 (18). Besides elder age, considered a key factor in the proposed clinical severity risk score, obesity and diabetes with complication had the highest COVID-19 death risk ratio of 1.3 among comorbid conditions, followed by chronic kidney disease, chronic obstructive pulmonary disease, and neurocognitive disorders (Death risk ratio of 1, 2). The existence of multiple comorbidities was reported as increasing the risk of COVID-19 mortality. Noteworthy that obesity was not mentioned in preliminary reports in China among the most common comorbidities predisposing for COVID-19 infection and COVID-19 disease severity, which was later attributed to the lower rates of obesity seen in far-east cultures (28). This underscores the need for consideration of the epidemiological state of one country for effective planning and the establishment of scoring systems.

Considering the criteria included in the prioritization system developed in this study, “heart condition/insufficiency,” “respiratory insufficiency,” “bone marrow or organ transplantation”, had scores between 10.8 and 10.1 %, followed by “immunodeficiency,” “diabetes,” and “renal failure” (9.6, 9, and 8.4%, respectively). “High blood pressure” and “evolutive cancer” were the least important criteria for the experts on average. When comparing these found scores to the risk ratio relative to each comorbidity in COVID-19 severity or fatality as reported in cohort studies, we encountered two limiting factors. First, the comorbidity to assess is not preponderant in the corresponding population (as obesity in China). Second, patients may present multiple comorbidities that may be correlated (exp: diabetes and obesity) resulting in multicollinearity in regression analysis. Within QCOVID, a coronavirus risk prediction model used to support the NHS coronavirus response in England, the risk ratios of hospital admission associated with the following factors, chronic kidney stage 4, solid organ transplant, and type 2 diabetes, were, respectively, 1.34, 1.79, 1.57, and 2.64 (Adjusted Hazard ratios for body mass index (BMI) and age). There was a high variability of the ratios found for different stages of kidney failures or with the type of diabetes (29).

Including more levels and more precision in the severity or types of disease in our study, as adding different stages of renal failure, types of diabetes or complicated diabetes, the severity of hypertension, would have resulted in increased granularity in the scoring of these comorbidities and could be considered as a limitation of our study.

To the best of our knowledge, this is the first time MCDA was applied to prioritize candidates for COVID-19 vaccination. This approach, by capturing and representing the “experts” preferences, is a rapid and innovative way to support decision-making protocols especially in the context of a lack of guidelines and limited available resources.

## Data Availability Statement

The original contributions presented in the study are included in the article/supplementary material, further inquiries can be directed to the corresponding author/s.

## Ethics Statement

Ethical review and approval was not required for the study on human participants in accordance with the local legislation and institutional requirements. Written informed consent for participation was not required for this study in accordance with the national legislation and the institutional requirements.

## Author Contributions

HC, MA, and MK conceived the study. HC and AR worked on the design of the survey, processed MCDA model steps and used 1,000 minds software and STATA. HC, MA, IZ, AH, and MK reviewed the literature and the national committees to decide the final criteria to include and score in the study. HC, AR, and IZ drafted the manuscript. MA and MK supervised the research steps. All authors have read and agreed to the published version of the manuscript.

## Conflict of Interest

The authors declare that the research was conducted in the absence of any commercial or financial relationships that could be construed as a potential conflict of interest.

## Publisher's Note

All claims expressed in this article are solely those of the authors and do not necessarily represent those of their affiliated organizations, or those of the publisher, the editors and the reviewers. Any product that may be evaluated in this article, or claim that may be made by its manufacturer, is not guaranteed or endorsed by the publisher.
